# Thyroid antibodies: the end of an era?

**DOI:** 10.1093/braincomms/fcab030

**Published:** 2021-03-10

**Authors:** Stefanie J Rodenbeck, Stacey L Clardy

**Affiliations:** 1 Department of Neurology, University of Utah, Salt Lake City, UT 84108, USA; 2 George E. Wahlen Veterans Affairs Medical Center, Salt Lake City, UT 84148, USA

**This scientific commentary refers to ‘Brain dysfunction and thyroid antibodies: autoimmune diagnosis and misdiagnosis’, by Valencia-Sanchez *et al.* (https://doi.org/10.1093/braincomms/fcaa233).**

Since the landmark article in 1966 by Lord Brain et al.,^1^ Hashimoto encephalopathy (HE) has been a consideration for countless cases of altered mental status in association with elevated thyroid antibodies [i.e. thyroid peroxidase (TPO) and/or thyroglobulin]. In the general population, elevated TPO antibody titres can be detected in ∼13% of healthy individuals, and in more than a quarter of women over the age of 65 years.^2^ The steroid responsive nature of the HE was further described in 2006 by Castillo *et* *al.*,^3^ who coined the term ‘steroid-responsive encephalopathy associated with autoimmune thyroiditis (SREAT)’, specifying the diagnostic triad of encephalopathy, thyroid autoimmunity and improvement in symptoms with steroid treatment.

In 2020, Mattozzi *et* *al.*^4^ reported the poor disease specificity of the TPO antibody, clearly demonstrating that detection of TPO antibody positivity rate was similar amongst groups with suspected autoimmune encephalitis compared to control patients. As such, they concluded that it was imperative to rule out neural autoantibody positive encephalitis when considering a diagnosis of encephalopathy due to an elevated TPO antibody titre. The article in this issue by Valencia-Sanchez *et* *al.* includes an even more expansive patient population and further adds to the data arguing against the use of thyroid autoantibody testing to directly support a diagnosis of autoimmune encephalopathy.

Prior to the widespread availability and accuracy of neural autoantibody testing, thyroid antibodies were used to support evidence of an autoimmune basis for encephalopathy in patients. In addition to mental status changes, patients with elevated thyroid antibodies were also described to have lateralizing motor dysfunction, movement disorders, seizures, and psychiatric symptoms. Subsequent response to immunotherapy, specifically steroids, would overwhelmingly result in a patient being diagnosed with HE/SREAT. Many times, additional evaluation with electroencephalography (EEG), imaging, neurocognitive testing and serological and CSF monitoring were not undertaken. The risk of misdiagnosis without an appropriately comprehensive investigation is 2-fold: potentially unnecessary exposure to the risks of immunosuppression, and further, lack of access to appropriate treatments.

Valencia-Sanchez et al.^5^ address the tendency within autoimmune neurological workup to include thyroid antibody testing, which may lead to costly and precarious immunotherapy trials. This retrospective review over a 13-year period identified 144 patients referred to the Mayo Clinic for suspected HE/SREAT. After a clinical evaluation and review of serologic, CSF, imaging, neuropsychological and EEG testing, 39 patients (27%) were eventually diagnosed with an autoimmune CNS disorder of whom 36 were seronegative for neural autoantibodies; diagnosis for seronegative patients was determined using criteria described by Graus et al.^6^ in 2016, and in this cohort, the sensitivity of such criteria was 92% and the specificity was 100%. Of the remaining 105 patients, final diagnoses most often included functional disorders, neurodegenerative disorders, subjective cognitive changes, chronic pain syndrome, primary psychiatric disorders and sleep disorders. These patients, as compared to those with autoimmune CNS disorders, were more likely to have symptoms of depression (*P* = 0.008), anxiety (*P* = 0.003) and chronic pain (*P* = 0.002). The patients who eventually were given diagnoses of CNS autoimmune disorders were more likely to have seizures (*P* = 0.008), stroke-like episodes (*P* = 0.007), language deficit (*P* = 0.04) and ataxia (*P* = 0.02). Most importantly, the TPO antibody titre was not significantly different between those with autoimmune CNS disorders and those with other diagnoses: the median TPO antibody titre for those with a final diagnosis of an autoimmune CNS disorder was 312.7 IU/ml; for those patients with an alternative diagnosis, the median titre was 259.4 IU/ml, for a non-significant difference of *P* = 0.44. Patients with an autoimmune CNS disorder did have significant differences in objective findings including lower serum vitamin B12 levels (*P* = 0.001), abnormal magnetic resonance imaging (MRI) with features consistent with autoimmune encephalitis (*P* = 0.003), abnormal findings on EEG (*P* = 0.007), and evidence of inflammation on cerebrospinal fluid examination (*P* = 0.002).^5^

Prior to quaternary referral centre evaluation, for patients analysed in the manuscript, 110 of the 144 (76%) patients received a trial of immunotherapy. Patients who eventually received a diagnosis of autoimmune CNS disease reported clinical improvement more frequently than those ultimately diagnosed with another condition (*P* < 0.001). While at the Mayo Clinic, additional trials of immunotherapy were given for 24 autoimmune CNS disorders patients, and 16 of these patients had demonstrable objective improvement. Improvement could be objectively demonstrated by using mental status testing, neuropsychological testing and MRI. Of the 12 patients with an alternative diagnosis who received an additional trial of immunotherapy, none had objective improvement.^5^

As discussed in this article, the duality of diagnostic possibilities of symptom aetiology as being autoimmune or not is myopic at best. A diagnosis of autoimmune CNS disease based solely on thyroid antibodies, however, is equally dangerous for patients. Evidence of systemic autoimmunity can raise suspicion for association with encephalopathy, but additional investigation must be undertaken. An approach of empiric steroids for uncertain aetiology of encephalopathy without data suggestive of an autoimmune aetiology beyond thyroid antibodies, especially when lacking other supportive objective data, is arcane and is not in the best interest of the patient. Application of established consensus guidelines for diagnosis of possible and probable autoimmune encephalitis allow for more appropriate trials of immunotherapy.^5^ Utilization of objective data to demonstrate improvement, as measured before and after a pre-specified duration of a diagnostic treatment trial, is paramount to inform consideration for future or ongoing immunotherapy, but similar to other neural autoantibodies,^7^ there is no indication to trend thyroid autoantibody titres.

In the interest of our patients, we must recognize the new evidence that demonstrates that the diagnostic utility of thyroid antibody testing is limited in the evaluation of autoimmune neurologic conditions. Its role is, at best, limited to demonstrating a potential propensity to systemic autoimmunity. Cessation of thyroid antibody testing in the evaluation of suspected autoimmune encephalitis and encephalopathy will hopefully reduce harm from misdiagnosis and unwarranted treatment, and minimize financial toxicity related to unnecessary interventions.

## Data Availability

Data sharing is not applicable to this article as no new data were created or analysed.
